# Key Imaging Factors for Transcatheter Management of Tricuspid Regurgitation: Device and Patient Selection

**DOI:** 10.3390/jcm13206144

**Published:** 2024-10-15

**Authors:** Francesco Cannata, Kamil Stankowski, Michele Galasso, Manuela Muratori, Elisabetta Mancini, Antonio Colombo, Gianluca Pontone, Federico De Marco, Fabio Fazzari, Antonio Mangieri

**Affiliations:** 1 Department of Perioperative Cardiology and Cardiovascular Imaging, Centro Cardiologico Monzino IRCCS, 20138 Milan, Italygianluca.pontone@cardiologicomonzino.it (G.P.);; 2 IRCCS Humanitas Research Hospital, Via Alessandro Manzoni, 56, Rozzano, 20089 Milano, Italy; 3 Department of Biomedical Sciences, Humanitas University, Via Rita Levi Montalcini, 4, Pieve Emanuele, 20090 Milano, Italy; 4 School of Medicine and Surgery, Milano-Bicocca University, 20126 Milan, Italy; m.galasso7@campus.unimib.it; 5 Department of Biomedical, Surgical and Dental Sciences, University of Milan, 20122 Milan, Italy; 6 Department of Interventional Cardiology, Centro Cardiologico Monzino IRCCS, 20138 Milan, Italy

**Keywords:** tricuspid regurgitation, structural interventional cardiology, transcatheter edge-to-edge repair, transcatheter tricuspid valve replacement, orthotopic valve replacement, heterotopic valve replacement, interventional echocardiography, interventional imaging, structural heart imaging, multimodality imaging

## Abstract

The growing awareness of tricuspid regurgitation (TR) and the fast-expanding array of devices aiming to percutaneously repair or replace the tricuspid valve have underscored the central role of multi-modality imaging in comprehensively assessing the anatomical and functional characteristics of TR. Accurate phenotyping of TR, the right heart, and pulmonary vasculature via echocardiography, computed tomography, and, occasionally, cardiovascular magnetic resonance and right heart catheterization is deemed crucial in choosing the most suitable treatment strategy for each patient and achieving procedural success. In the first part of the present review, key imaging factors for patient selection will be discussed. In the ensuing sections, an overview of the most commonly used, commercially available systems for transcatheter repair/replacement will be presented, along with their respective selection criteria and information on intraprocedural imaging guidance; these are edge-to-edge repair, orthotopic and heterotopic replacement, and valve-in-valve procedures.

## 1. Introduction

Tricuspid regurgitation (TR) is a prevalent valvular heart disease that significantly impacts prognosis and quality of life [1,2,3]. Moderate TR has a prevalence of at least 0.55% in the general population [1], reaching a prevalence as high as 7.2% in the older population (≥65 years) in a recent community-based study [4]. Compared to patients with no valvular heart disease, those with TR had a 2.5 higher risk of death in a large UK Biobank cohort [5]. Another large study confirmed the association of increasing TR severity with adverse outcomes, suggesting that even mild TR has a significant prognostic impact [6]. In recent years, the importance of prompt recognition and treatment of TR has been acknowledged and intense research work has worked to clarify important aspects of TR diagnosis, its pathophysiology, and its prognostic impact in diverse TR populations [7,8,9]. The latest 2021 ESC/EACTS valvular heart disease guidelines recommend that interventional treatment of secondary TR is considered in experienced centers in anatomically eligible symptomatic patients who are not deemed good surgical candidates [10]. Meanwhile, advances in surgical techniques and risk stratification have led to a reappraisal of tricuspid valve (TV) surgery, once considered high-risk. The TRI-SCORE incorporates eight clinical and imaging parameters and can predict in-hospital mortality following isolated tricuspid surgery, assisting in the clinical decision-making process [11]. Nonetheless, a significant proportion of patients with TR are elder and comorbid and thus unfit for (re-do) surgery [12], whereas tricuspid transcatheter valve intervention (TTVI) is a safe option in high-TRI-SCORE-category patients [13]. Recently, a dedicated risk score (TRIVALVE score) has been developed specifically for this population [14].

The array of percutaneous options is constantly expanding, and, among the commercially available options, a prominent role is currently occupied by transcatheter edge-to-edge-repair (TEER) and, at a distance, orthotopic and heterotopic valve replacement. The availability of different devices underscores the need for an accurate preoperative imaging assessment in order to choose the most appropriate device according to individual anatomical and clinical characteristics, taking into consideration local expertise and availability. Moreover, most of the devices require expert intraprocedural guidance for procedural success [15]. In the present narrative review, we discuss available evidence (mostly observational) regarding TTVI and authors’ accumulated experience in an attempt to provide a comprehensive yet practical guide for clinicians, despite not using a systematic approach for literature search. In the following sections, an overview of the imaging factors to be considered prior to tricuspid percutaneous repair/replacement and the specific characteristics of the most utilized devices will be presented.

## 2. Key Imaging Factors for Patient Selection

### 2.1. Tricuspid Valve Anatomy and Remodeling

**Figure 1:**
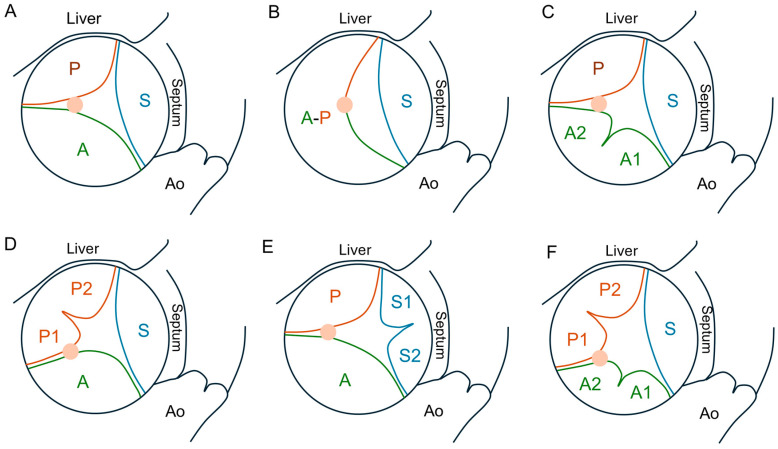
Tricuspid leaflet anatomy classification. (**A**) Tri-leaflet anatomy. (**B**) Bicuspid anatomy with a septal leaflet and a large anteroposterior leaflet. (**C**–**E**) Four-leaflet anatomy with a bipartite anterior, posterior or septal leaflet, respectively. (**F**) Five-leaflet anatomy with bipartite anterior and posterior leaflets. Abbreviations: A = anterior leaflet; P = posterior leaflet; S = septal leaflet; Ao = aorta. The pink circle denotes the position of the anterior papillary muscle.

The TV is the largest cardiac valve with an orifice that normally ranges between 7 and 9 cm^2^ [16], sitting between the right atrium (RA) and the right ventricle (RV) and normally allowing passage of blood in diastole and preventing regurgitation in systole. The proper function of the TV is dependent on the leaflets, papillary muscles, annulus and chordae. The TV normally presents three leaflets (anterior, posterior and septal); however, compared to its left-sided counterpart, the TV shows significant morphological variability in terms of leaflet/scallop number and size. In the most common setting, the anterior leaflet is the biggest, and the posterior leaflet is the smallest. Hahn et al., in a seminal trans-esophageal echocardiography (TEE) study [17], established a standard for a universal nomenclature of the TV. It was found that a tricuspid morphology was present in 54% patients, and 5% had a bicuspid morphology, whereas the remaining patients had a four-leaflet morphology (with a bipartite posterior leaflet being the most common, at 32%) or, rarely, a five-leaflet morphology (Figure 1).

Recognizing the individual morphology of the TV in the preoperative assessment is especially important for TEER as the variability of number and location of supernumerary leaflets may contribute to procedural failure. Notably, a four-leaflet configuration has been associated with increased risk of residual TR [18]. A recent autopsy study confirmed a high variability in leaflet patterns and found the most common configuration to be non-scalloped anterior and septal with scalloped posterior [19]. Compared to the mitral valve, normal TV leaflets are much thinner, which makes them more difficult to image via echocardiography [20], and poor echocardiographic leaflet visualization is a relative contraindication to TEER. High-quality multiplane TEE imaging is, in fact, necessary for the evaluation of complex leaflet anatomy and regurgitant jets and monitoring of the grasping process [21].

The TV papillary muscles can be distinguished into anterior (the most prominent and typically fused with the moderator band), posterior, and septal, despite there being many variants [22]. The anterior papillary muscle is a landmark for recognizing TV leaflet anatomy as it marks the anteroposterior commissure; the anteroseptal commissure sits adjacent to the aortic valve, whereas the posteroseptal commissure is next to the coronary sinus [17,23]. Dilatation of the RV may apically displace the anterior papillary muscle causing ventricular tethering of the anterior and posterior leaflets in the systole, whereas altered septal position or mobility may affect the septal leaflet [24].

Rather than a well-defined anatomic structure, the TV annulus is an oval and saddle-shaped functional unit [25]. Annular size changes throughout the cardiac cycle, being largest at the end-diastole [26]. The normal tricuspid annular circumference and area are 12 ± 1 cm and 11 ± 2 cm^2^, respectively [27,28]. With TR, the annulus becomes larger, more planar, and circular [28]. Annular dilatation is a feature of secondary atrial TR or long-standing TR of any type and occurs along the attachments of the anterior and posterior leaflets because the septal portion is rigidly attached to fibrous skeleton of the heart [16]. Consequently, the regurgitant orifice has usually an elliptical shape with the greatest length from anterior to posterior [29]. The tricuspid leaflets physiologically have an excess coaptation length of 5 to 10 mm [22], and some degree of annular dilatation (up to 40% of the TV annulus area) may occur before significant regurgitation ensues [30]. In an attempt to restore a physiological leaflet-to-annulus ratio and decrease TR severity, compensatory leaflet growth by means of endothelial–mesenchymal transition has been described [31]. Variability exists in the development of atrial functional TR in patients with atrial fibrillation owing to different degrees of compensatory leaflet growth, different number and morphology of the leaflets, and varied right atrial function [17].

Preprocedural imaging of the TV relies on a multimodality approach including transthoracic echocardiography (TTE), TEE, cardiac computed tomography (CT), and, occasionally, cardiac magnetic resonance (CMR) to assess TV morphology, quantify TR, evaluate the right heart, pulmonary pressures, and access routes. Specific structural imaging is dependent on the planned type of device to be used; however, a precise definition of the etiology and mechanism of TR is a prerequisite of any intervention and one of the main factors that dictates eligibility for a given transcatheter repair/replacement strategy [21]. A thorough preprocedural imaging assessment is also needed for planning valve-in-valve implantation in a failing tricuspid bioprosthesis to confirm the degree of regurgitation and/or stenosis and rule out paravalvular leaks, endocarditis, or thrombosis. Regarding TR etiology, contemporary classification schemes include primary (or organic) TR, secondary (or functional) ventricular TR, secondary (or functional) atrial TR, and CIED-related TR [25] (Table 1).

The atrial phenotype of secondary TR has been recently recognized, with predominant dilation of the RA and tricuspid annulus, commonly occurring in elderly women with atrial fibrillation and/or heart failure with preserved ejection fraction, although there are some overlapping features with the ventricular phenotype, especially in long-standing disease [31]. Recently, the first Tricuspid Valve Academic Research Consortium (TVARC) consensus paper has been published with the aim to standardize definitions of TR etiology and severity and define consistent endpoints for tricuspid trials to allow for meaningful comparisons among them [32].

Although TTE is, in most cases, sufficient to grade the severity of TR and assess the etiology of TR, TEE is indispensable for planning TEER. The TEE study of the TV is complicated by its anterior, far-field position in the imaging sector, suffering from acoustic shadowing by mitral and aortic valves, especially when calcified or in the presence of prosthetic valves, as well as the shallow angles of insonation, and the need for larger 3D volumes [24]. As such, multiple dedicated imaging windows (mid-esophageal, deep-esophageal, transgastric and deep-transgastric) are needed [16,23]. Several landmarks are of help in identifying individual leaflets, although due to the variability in leaflet position/size, biplane imaging should be used to confirm which leaflet is visualized, and the transgastric view—allowing simultaneous en-face visualization of all the three (or more) leaflets—should be always obtained [33]. Independent motion of the leaflet motion and systolic color flow around the leaflet edges are useful in differentiating supernumerary leaflets from minor clefts. Three-dimensional echocardiography, usually acquired at the imaging window where imaging quality is best, has significantly improved the accuracy of identification of the leaflets and is an essential part of preprocedural assessment.

**Figure 2:**
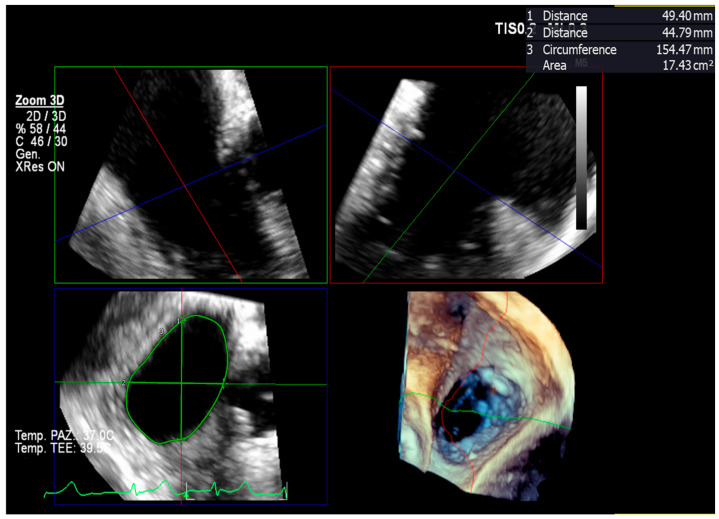
Reconstruction of the TV annulus from a 3D dataset with indicated the antero-posterior and septo-lateral diameters, annular circumference and area.

The TV annulus can be reconstructed from a 3D dataset, and the septo-lateral and antero-posterior diameters are indicated (Figure 2).

Notably, 2D measurements are systematically underestimated [34]. Cardiac CT is able to provide high quality images of the tricuspid annulus with excellent spatial resolution and good temporal resolution using a dedicated protocol for optimal right heart opacification [23,35]. The tricuspid annular plane can be reconstructed using multiplanar reconstructions with a reliable assessment of the septo-lateral and antero-posterior diameters, perimeter, and area at 5–10% increments of the R-R interval. The annulus can be traced manually or using semi-automated software; however, the agreement between TEE and CT for annulus sizing was found to be superior using semi-automated methods [36]. Although 3D echocardiographic imaging is accurate, CT sizing is generally preferred for orthotopic valve replacement feasibility and sizing studies. In addition, cardiac CT allows assessment of valvular and subvalvular morphology and vascular access routes for preprocedural planning. Cardiac CT is also essential for heterotopic valve replacement in order to assess superior and inferior venae cavae dimensions and the risk of hepatic vein obstruction [37] and confirm the size of an existing bioprosthesis in cases of valve-in-valve procedures, if not known [21]. On the other hand, CT is generally not necessary for TEER.

### 2.2. Coaptation Gap and Tricuspid Regurgitation Severity

The quantification of TR necessitates a multiparametric approach integrating multiple quantitative, semiquantitative, and qualitative measures, as described in the European Association of Cardiovascular Imaging and American Society of Echocardiography guidelines [38,39]. Echocardiography remains the reference technique. Importantly, preprocedural evaluation of TR severity should be carried out after optimization of volemic status, restoration of sinus rhythm, or adequate rate control [40]. The severity of TR is graded in five tiers: mild, moderate, severe, massive and torrential (Table 2).

The addition of massive and torrential grades stems from the observation that the previous classification in four degrees (up to severe) failed to account for patients with “very severe” regurgitation that could still benefit from transcatheter intervention [41]. Notably, patients with massive or torrential TR have worse outcomes in terms of mortality and heart failure hospitalization than those with severe TR [42]. Not surprisingly, baseline massive or torrential TR has been associated with reduced likelihood of achieving moderate or less TR after transcatheter tricuspid valve intervention (TTVI). In the TRILUMINATE trial, only 53% of patients with torrential and massive TR at baseline achieved moderate or less TR at follow-up, whereas the majority (90%) of them had at least one grade reduction in their baseline TR severity [43]. However, in the bRIGHT registry, where 88% of the included patients had massive or torrential TR at baseline, moderate or less residual TR was achieved in 85% of the patients at 30-day follow-up and 81% at one-year. Residual moderate or less TR was associated with lower one-year mortality [44].

The contemporary expanded scheme has been reappraised by the TVARC consensus [32]. Dreyfus et al. have recently advocated for a new TR severity classification differentiating the moderate-degree group into mild-to-moderate and moderate-to-severe subgroups, due to a significantly different prognosis, resulting in a four-degree classification (mild, mild-to-moderate, moderate-to-severe, and severe) similar to the mitral regurgitation grading or a six-degree classification if incorporating the massive and torrential grading [45,46].

If echocardiography is inconclusive, CMR can be used as an adjunct method for TR quantification. Apart from being the reference method for RV volume and function quantification, CMR is able to directly measure flow through phase contrast sequences and derive a precise estimate of the tricuspid regurgitant volume and regurgitant fraction [47]. Several echocardiographic parameters used for grading TR have demonstrated good accuracy against regurgitant volume by CMR [48]. Four-dimensional flow imaging, a novel technique allowing comprehensive evaluation of flow inside the heart and blood vessels in all three spatial dimensions within the acquired volume, holds promise for further refining TR quantification by CMR [49]. In a study, four-dimensional flow evaluation of TR severity showed good reproducibility and correlation with conventional CMR assessment using the direct and indirect methods [50].

**Figure 3:**
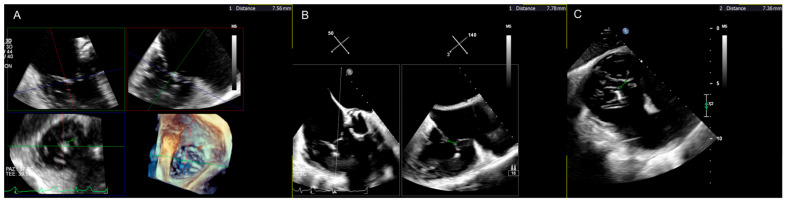
Coaptation gap measurement on a multiplanar reconstruction of a 3D trans-esophageal echocardiography dataset – red, blue and green lines represent orthogonal reference planes (**A**); biplane commissural view (**B**); transgastric en-face view (**C**).

Apart from establishing TR severity, preprocedural imaging assessment also determines the feasibility and probability of the success of specific techniques, such as TEER [15]. In this regard, it is crucial to assess the coaptation gap, defined as the largest systolic distance between the septal and the anterior or posterior leaflet tips at the proposed grasping zone [51], as grasping typically occurs in that direction. Larger coaptation gaps are more challenging (or impossible) to target with an edge-to-edge device and may result in the clip being placed away from its primary target. They have been associated with a higher risk of residual regurgitation. In a study, 77% of patients with coaptation gaps <7 mm achieved moderate or less TR, compared to 43% and 33%, respectively, for patients with coaptation gaps of 7–10 mm and >10 mm. The optimal cut-off for predicting procedural success was established at <8.4 mm. In another study, smaller coaptation gaps were predictive of procedural success (with an optimal cut-off of <7.2 mm) [52]. Similar results were reported by the echocardiographic analysis of the bRIGHT registry [53]. On the contrary, a long gap in the anteroposterior direction but with a reduced coaptation gap is conducive to successful implantation of more devices. The coaptation gap is a load-dependent and dynamic parameter that varies according to the volemic status of the patient. The coaptation gap can be measured from a reconstructed 3D dataset or 2D images (Figure 3).

Adequate visualization of leaflet tips is in any case necessary to avoid overestimation of the coaptation gap, which is especially frequent if evaluated on the 2D transgastric en-face view [53]. The location of the coaptation gap that is intended to be addressed is linked to procedural outcomes, as non-anteroseptal/central coaptation gaps have been associated with greater risk of residual TR [18,51,52] because grasping in the posterior region is technically more challenging. Moreover, grasping in the anteroseptal position has been associated with more pronounced reduction of the tricuspid annulus diameter [54].

Additional considerations for TEER are the adequacy of leaflet length and tissue quality and the degree of leaflet tethering which can be expressed as tethering height (perpendicular line from the coaptation point to the annular plane) or tenting area (enclosed between the annular plane and the coaptation point). Extreme leaflet tethering precludes effective grasping, and a tenting area >2.1 cm^2^ was associated with procedural unsuccess [52]. In cases of significant annulus dilatation, an imbalance of the leaflet length to annulus area ratio has been associated not only with development of functional TR but also with significant residual regurgitation after TEER [55,56].

Because the TV annulus is much larger than the mitral annulus, tricuspid stenosis is rare after TEER, and gradients have not been associated with outcomes following TEER [57]. On the other hand, it can occur after transcatheter valve replacement due to structural valve dysfunction, endocarditis, or leaflet thrombosis. Tricuspid mean and maximum gradients, pressure half-time, the diastolic velocity–time integral, and the direct planimetry of the tricuspid orifice on reconstructed 3D datasets can be assessed.

### 2.3. Right Heart Remodelling

A comprehensive assessment of right heart remodeling is pivotal for the understanding of TR etiology, valve remodeling mechanisms, and patient prognosis. The diagnostic evaluation of the RV has posed several challenges due to its morphology, its limited echocardiographic parameters, and the reduced evidence of its prognostic impact. The recent advancements in imaging techniques and the growing focus on TR have expanded the available data.

The evaluation of right heart remodeling parameters is fundamental to distinguish between ventricular and atrial functional TR [58,59]. As widely demonstrated and incorporated by TVARC, atrial functional TR patients exhibit a larger RV basal diameter and volume, with a conical RV remodeling characterized by elevated basal-to-mid and basal-to-longitudinal diameter ratios and a diminished sphericity index and a mildly diminished RV systolic function compared to healthy controls. Meanwhile, ventricular functional TR patients display significantly abnormal RV dimensions and volumes—with an RV spherical or elliptical deformation—due to increased ratios across all diameters and increased sphericity index coupled with markedly reduced RV systolic function [32]. Both types of functional TR show considerable dilatation of the RA, with notably larger minimum RA volumes in atrial compared to ventricular TR. Valvular tethering, tenting height, and volume are significantly elevated in ventricular functional TR compared to atrial functional TR patients. On this basis, the onset of a significant TR may induce further remodeling alterations as TR causes right heart volume overload, resulting in dilatation of the RA, tricuspid annulus, and RV. This triggers further papillary muscle displacement and alters right cardiac dynamics, exacerbating TR and initiating a detrimental cycle marked by progressive RV dilation, dysfunction, and clinical decline. Hence, an advanced atrial functional TR may eventually resemble a ventricular functional TR [60], as right heart remodeling is a dynamic process which may sometimes be inadequately described by rigid classifications.

Regarding the prognostic impact of the underlying etiology of functional TR, Bombace et al. [59] recently showed that in patients with significant TR undergoing different treatment options, RV dysfunction, identified by reduction of tricuspid annular plane systolic excursion (TAPSE) and fractional area change (FAC), was associated with higher mortality, whereas increasing RA and tricuspid annulus dilation were associated with reduced mortality. These findings provide further support for the hypothesis that atrial functional TR may have a more favorable prognostic outcome compared with ventricular functional TR. Therefore, a thorough imaging assessment provides prognostic clues which may help to identify those patients who will most benefit from TTVI. Recently, Tanaka et al. [61] highlighted the prognostic importance of post procedure changes in RV function in a cohort of 204 patients with symptomatic severe TR undergoing TEER. Baseline RV-FAC < 35% was linked to higher risk of mortality and heart failure hospitalization at one year. Improvement in FAC post procedure reduced the risk, especially in patients with baseline FAC < 35%. Smaller RV diameter and greater TR reduction predicted RV function improvement, underscoring that TEER should not be denied to patients with preprocedural RV dysfunction based solely on reduced RV-FAC. Previous studies had struggled to demonstrate the predictive power of conventional echocardiographic parameters for clinical outcomes in tricuspid valve repair [62,63]. Traditional 2D echocardiographic measures—TAPSE, S’ and FAC—are influenced by geometric assumptions, loading conditions, and imaging angles, failing to capture the entire RV contraction. FAC neglects the RV outflow tract, while TAPSE and S’ ignore the contribution of the RV free-wall and septum. The apical four-chamber view introduces variability, making RV dimensions inconsistent [64]. CMR and 3D echocardiography are considered gold standards as they do not rely on geometric assumptions and can directly measure RV ejection fraction (RVEF). The RV unique 3D structure, characterized by a crescent-shaped muscular band, is not adequately represented by 2D parameters [65,66]. Severe TR-induced volume overload can further distort fiber orientation, and 3D echocardiography in this setting provides more precise measurements of RV volumes and ejection fraction compared to 2D echocardiography [67]. In a study involving 47 patients undergoing both 3D echocardiography and CMR, Muraru et al. [68] demonstrated that automated software for post-processing 3D echocardiographic data exhibited excellent concordance with CMR imaging for measuring RV end-diastolic volume (bias 5 ± 24 mL) and ejection fraction (bias 1.4 ± 4.9%).

However, RVEF solely reflects volume change without considering flow direction. Consequently, conventional echocardiographic parameters may overestimate RV function in cases of severe TR with subclinical dysfunction. RV free-wall longitudinal strain (RVFWLS) has emerged as a valuable tool for assessing RV function, detecting higher rates of dysfunction, and predicting adverse outcomes beyond conventional parameters in patients with significant TR [69]. Ancona et al. [70] demonstrated that the use of RV strain may identify the presence of systolic dysfunction, which was not evident with traditional parameters. Indeed, in a population of 250 consecutive patients with severe TR, the use of both RVFWLS and RV global longitudinal strain led to the reclassification of 42–56% of patients initially categorized as having normal RV systolic function based on traditional parameters. An RVFWLS value above −17% was predictive of the presence of right ventricular heart failure, while patients with RVFWLS below −14% exhibited better survival rates at follow-up. Measurement of RVEF can be conducted using various imaging techniques (CMR, 3D echocardiography, cardiac CT), but it fails to consider the interplay between RV contractility and afterload. Consequently, it may overstate RV systolic function in cases of severe TR. Various invasive and non-invasive parameters exist for evaluating ventricular–arterial coupling, recently validated by several studies [71,72]. Brener et al. [73] examined RV–pulmonary artery (PA) coupling ratios in patients enrolled in the global TriValve registry [74]. They calculated the TAPSE divided by the PA systolic pressure (PAP) from transthoracic echocardiograms conducted pre- and post-procedure. Among the 444 patients analyzed, the median TAPSE/PAPs ratio stood at 0.406 mm/mmHg. Within one year of TTVI, 63 patients died, 21 with a TAPSE/PAPs ratio above 0.406 and 42 with a ratio below 0.406. Multivariable Cox regression analysis revealed that a TAPSE/PAPs ratio above 0.406 was linked to a reduced risk of all-cause mortality. Among the 234 patients (52.7%) who underwent echocardiograms 30 days post-TTVI, a decline in RV-PA coupling correlated independently with decreased odds of all-cause mortality. Moreover, the extent of TR reduction post-TTVI was independently associated with a decrease in RV-PA coupling following TTVI [73].

Cardiac CT can overcome several limitations of 2D echocardiography by offering superior anatomical and functional assessments of the TV apparatus and its surrounding structures, hence it is vital for screening and preprocedural planning of transcatheter TV replacement but is not mandatory for T-TEER [20,75]. Cardiac CT can also offer valuable insights into right chambers, such as RV and RA volumes, RVEF, and stroke volume [75,76,77]. For patients with intracardiac leads that are not CMR-compatible, CCT is a practical alternative for right chamber evaluation and can also evaluate how TV leaflets interact with intracardiac leads when echocardiography is insufficient [15,78].

CMR is not the primary imaging technique, but due to its excellent spatial resolution it adds value to 3D echocardiography for both anatomical and functional evaluation of the TV, tricuspid annulus, and right-sided chambers [79]. CMR is beneficial for assessing the severity of regurgitant TV lesions in cases with poor echocardiographic quality or conflicting results and serves as the reference method for evaluating RV volumes and function, enabling an assessment of the impact of TR and myocardial fibrosis [80]. While specific CMR thresholds for TR severity have not been established, a regurgitant fraction of 40% or more is typically considered hemodynamically significant [78]. Functional TR severity, as determined by CMR, is an independent predictor of mortality, even after adjusting for clinical and imaging variables, including RVEF. A TR volume of 45 mL or more or a TR fraction of 50% or more is associated with the highest risk of excess mortality [81]. Preprocedural CMR-derived RVEF and RV end-systolic volume are independently linked to increased postoperative cardiac death and major cardiac events in patients undergoing corrective surgery for isolated severe TR [81]. In one study [82], global RV dysfunction was defined as a CMR-derived RVEF of less than 45%, and longitudinal RV dysfunction was defined as TAPSE of less than 17 mm on echocardiography. Patients were categorized into three types of RV contraction: type I (TAPSE > 17 mm and RVEF > 45%), type II (TAPSE < 17 mm and RVEF > 45%), and type III (TAPSE < 17 mm and RVEF < 45%). CMR feature tracking was used to assess longitudinal and circumferential RV strain. Among 79 patients, 18 (23%) had global and 40 (51%) had longitudinal RV dysfunction. Global, but not longitudinal, RV dysfunction was associated with the composite outcome. Compared to type I RV contraction, patients with type II RV contraction showed increased circumferential strain, preserving RVEF despite reduced longitudinal strain. Patients with type III RV contraction exhibited decreased longitudinal and circumferential strain, leading to impaired RVEF. These patients had the poorest survival rates. CMR also provides a non-invasive measurement of pulmonary vascular resistance (PVR), aiding in the selection of candidates for tricuspid interventions, as patients with end-stage RV failure may have reduced pulmonary artery pressure but increased PVR [83]. Lastly, detecting myocardial fibrosis with CMR has prognostic significance for RV failure and can help determine the optimal timing for interventions in patients with severe TR [15].

### 2.4. Pulmonary Hemodynamics

**Figure 4:**
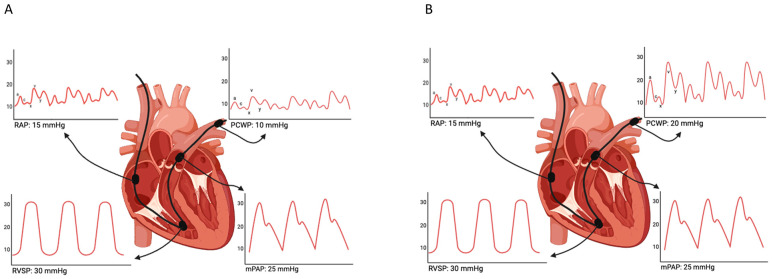
Right heart catheterization (RHC) in tricuspid regurgitation. (**A**) Example of RHC tracings in a case of TR in the absence of left heart disease. (**B**) Example of RHC tracings in TR secondary to left heart disease with elevated pulmonary capillary wedge pressure. Abbreviations: a: a-wave; c: c-wave; v: v-wave; x: x-descent; y: y-descent; RAP: right atrial pressure; PCWP: pulmonary capillary wedge pressure; RVSP: right ventricular systolic pressure; mPAP: mean pulmonary artery pressure.

Before performing TTVI, a comprehensive hemodynamic assessment with right heart catheterization (RHC) provides detailed information about right heart pressures, which helps in determining the severity of TR and the patient’s overall hemodynamic status [84]. TTE estimates pressures indirectly and relies on the quality of acoustic windows; this can result in poor image quality and incomplete assessment of cardiac structures and function. While TTE provides valuable qualitative information, such as the presence and severity of TR, it is less reliable in providing precise quantitative hemodynamic data compared to direct measurements. RHC provides direct and precise measurements of pressures within the RA, RV and PA. Using techniques such as thermodilution or the Fick principle, RHC can accurately quantify cardiac output (CO) [84]. RHC can directly measure pulmonary capillary wedge pressure (PCWP) and calculate PVR. These parameters are vital for evaluating the impact of TR on pulmonary circulation and for distinguishing between pre-capillary (pre-capillary-PH), isolated post-capillary (Ipc-PH) or combined post-and-pre-capillary pulmonary hypertension (Cpc-PH) [85]. In the context of mitral regurgitation, elevated left atrial pressure can lead to increased PCWP and arterialization of the pulmonary veins. This results in pulmonary hypertension, decoupling of the RV and PA, and eventually RV dilation and dysfunction. Concurrently, left atrium negative remodeling and enlargement occur, leading to the onset of atrial fibrillation, volume overload, and progressive enlargement of the RA. These processes collectively cause dilation of the tricuspid annulus, resulting in leaflet malcoaptation and TR [86]. Chronic elevation of pulmonary capillary pressure can impair endothelial function and permeability, leading to increased PVR, which exacerbates the vicious cycle of pulmonary hypertension (Figure 4).

Among the numerous parameters that can be assessed during RHC, few have been studied in the context of TTVI, leaving their prognostic impact largely unknown. In an international multicenter study [87] including 236 patients, TTVI reduced TR severity at discharge (grade ≥ 3 in 16% of patients), decreased the right atrial v-wave by 19% (from 21 mmHg to 16 mmHg), and enhanced CO (from 3.5 to 4.0 L/min). Key hemodynamic predictors of 1-year mortality included mean pulmonary artery pressure (PAPm), transpulmonary gradient (TPG), PVR, and right ventricular stroke work. Stratification by PAPm and TPG emerged as the best predictors of 1-year survival. Patients with predominantly pre-capillary-pulmonary hypertension had poor prognosis (1-year survival of 38%), whereas those without or with postcapillary pulmonary hypertension fared better (1-year survival of 92% and 78%, respectively). In another study [88] including 243 patients undergoing TTVI, patients were stratified according to both invasive and echocardiographic assessment of pulmonary pressures. Patients with an invasive diagnosis of pulmonary hypertension were at higher preoperative risk, had more severe symptoms, higher N-terminal pro-B-type natriuretic peptide levels, more impaired RV function, and afterload-corrected RV function. Echocardiography had a limited accuracy (55%) in detecting patients with invasively-confirmed pulmonary hypertension. Interestingly, the patients with a discordant diagnosis of pulmonary hypertension (invasive diagnosis of pulmonary hypertension not confirmed by echocardiography) had the worst prognosis, probably due to a more severe baseline TR determining the classical V-wave cut-off sign (i.e., a triangular continuous Doppler wave profile due to a rapid equalization of RA and RV pressures). On the other hand, patients without pulmonary hypertension and patients with concordant diagnosis of pulmonary hypertension had similar results. Hence, the presence of a discordant diagnosis of pulmonary hypertension may be used as a better exclusion criterion for TTVI than the echocardiographic diagnosis of pulmonary hypertension.

A new parameter analyzed in the context of TTVI is the pulmonary artery pulsatility index (PAPi), which measures the ratio of pulmonary artery pulse pressure to right atrial pressure, namely RV contractility against its afterload based on filling pressure. A study [89] analyzed PAPi and clinical outcomes after TTVI among 259 patients who underwent RHC before TTVI. The median PAPi was 2.2 (interquartile range: 1.5–3.4). Patients were categorized by PAPi levels: PAPi > 4 (49 patients), PAPi 2 to 4 (104 patients), and PAPi < 2 (106 patients). In-hospital mortality was comparable between the three groups, whereas the procedural success rate was lower in patients with lower PAPi levels (91.8% in PAPi > 4, 79.8% in PAPi 2 to 4, and 67.0% in PAPi < 2). Within 1 year after TTVI, 74 patients experienced the composite outcome. In comparison to patients with PAPi > 4, those with PAPi of 2 to 4 and <2 had higher rates of the composite outcome (15.5%, 34.5%, and 38.4%, respectively).

The TRILUMINATE trial excluded patients with PAPs > 70 mmHg at RHC [90]. There is need for research work to determine whether intervention is still beneficial in patients with severe pulmonary hypertension or in which subgroup. The presence of a significant discrepancy between echocardiographic and RHC parameters may be a key factor to select the right patients. Table 3 shows useful parameters for assessing the right heart and the pulmonary vasculature through different techniques.

### 2.5. CIED Leads

Cardiac implantable electronic device (CIED) leads are present in 20 to 35% of patients enrolled in transcatheter TV device trials [91]. The presence of transvalvular CIED leads across the TV is a demonstrated risk factor for TR progression. The first reports of an association between TR and CIED leads date back to 1998, when Paniagua et al. [92] detected a significantly higher prevalence of moderate-to-severe TR in patients with CIED leads (25%), as compared with the usual prevalence of TR in patients without CIED.

When approaching a patient with CIED leads and significant TR, it is imperative to define the type of CIED-TV interaction and to differentiate between a CIED-incidental TR (or CIED-associated TR), in which there is a “peaceful” coexistence, and a CIED-related TR in which there is a lead–leaflet interaction with a defined causality between lead and TR [93]. On this basis, CIED-related TR has been recently classified as a specific etiologic entity, as it requires a specific diagnostic work-up and shows different therapeutic challenges and options [32].

The mechanisms of CIED-related TR may be classified as follows [93].

- Leaflet-related: the CIED lead interferes with normal leaflet coaptation, directly or indirectly;
  ○ Direct interference.
    Leaflet impingement: the systolic leaflet excursion is limited by the interfering lead position;
    Leaflet adhesion: an adherent lead moves altogether with the leaflet throughout the whole cardiac cycle due to fibrotic connection;
    Leaflet perforation or laceration: an organic lesion induced during the lead positioning or extraction maneuvers.
  ○ Indirect interference:
    Leaflet avulsion due to CIED lead extraction or implantation;
    Lead-related endocarditis.
- Subvalvular apparatus-related: the interference regards the papillary muscles or chordae tendineae.
  Subvalvular entanglement/adhesion;
  Chordal rupture.
- Rhythm-related: the RV pacing-induced dyssynchrony may induce RV dysfunction and progressive TV remodeling with worsening TR.

As shown by Addetia et al., commissural and central trajectories are not generally associated with significant TR [94]. Moreover, there is a growing body of evidence investigating the impact of innovative pacing strategies (left bundle branch pacing, His bundle pacing, non-apical RV pacing, etc.) on CIED-lead related complications, including worsening TR [95]. In a study, leadless pacemaker implantation was associated with worsening TR in a minority of cases, and severe TR was present only in 4% of patients at one-year follow-up, making it an attractive pacing modality when avoidance of TR development or progression is highly desirable [96].

**Figure 5:**
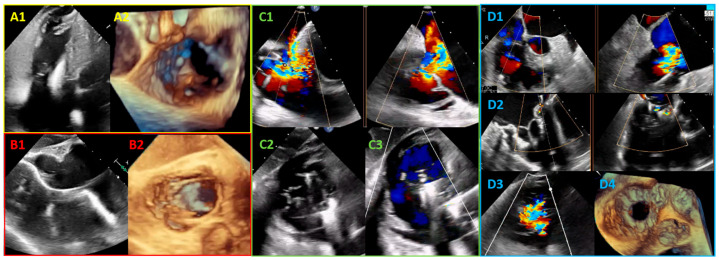
Tricuspid regurgitation and cardiac implantable electronic devices (CIED). (**A**) TTE RV-focused four-chamber window (**A1**) and 3D evaluation (**A2**) of a CIED-related TR due to septal leaflet impingement; (**B**) TEE transgastric long-axis window (**B1**) and 3D assessment (**B2**) of a CIED-related TR due to a spiral-shaped trajectory of CIED interfering with anteroseptal coaptation; (**C**) TEER with two clips in a case of CIED-incidental TR: baseline TEE inflow–outflow window with biplane-derived four-chamber view showing a functional atrial TR with a posterior CIED (**C1**), tricuspid transcatheter edge-to-edge repair with 2 TriClip devices in anteroseptal (**C2**) and posteroseptal (**C3**) positions; (**D**) tricuspid transcatheter valve replacement (TTVR) with a Cardiovalve device in a CIED-related TR due to posterior leaflet impingement: baseline TEE inflow–outflow window with biplane-derived four-chamber view (**D1**) and TEE transgastric short axis (**D3**) showing a CIED-related TR; optimal result of Cardiovalve TTVR procedure with minimal residual regurgitation assessed at 2D-TEE (**D2**) and 3D-TEE (**D4**).

The diagnostic assessment of the CIED-TV interaction is based essentially on echocardiographic imaging [91,93]. A complete TTE with multiwindow TV visualization may be sufficient for the diagnostic work-up [97]. However, TEE imaging is helpful in cases of suboptimal or insufficient TTE imaging for diagnosis and is necessary for therapeutic planning (Figure 5).

The TTE/TEE acquisition of 3D datasets and multiplanar reconstruction (in real time or in post processing) is particularly useful in defining the transvalvular trajectory of the lead and its relationship with TV components [23]. Moreover, the availability of longitudinal data regarding the evolution of TR before and after CIED implantation is helpful for diagnostic definition. It is noteworthy that the presence of CIED leads may induce an underestimation of the TR severity upon color-Doppler evaluation (above all with TTE) due to the acoustic impedance and reflectivity of the leads.

The integration of TTE/TEE with a CT scan is helpful for diagnostic purposes and pivotal for therapeutic planning [23,91]. The role of CMR is limited to myocardial tissue characterization, right heart dimensions, RV function, and TR quantification in cases of inconclusive echocardiographic evaluation. Moreover, CIED-related artefacts and the necessity of systematic device interrogation before and after CMR further reduce the application of this modality for this TR subtype.

Once multiwindow echocardiography and multimodality imaging have solved the diagnostic riddle of the causal role of CIED in a non-surgical patient with significant TR (CIED-incidental TR vs. CIED-related TR), the therapeutic options are the following [91]:

- transvenous lead extraction in CIED-related TR;
- tricuspid transcatheter edge-to-edge repair (T-TEER) in the presence of CIED-incidental TR and in selected cases of CIED-related TR, provided that TR is “graspable”;
- orthotopic tricuspid transcatheter valve replacement (TTVR) with lead jailing in presence of CIED-related TR or in cases of CIED-incidental and “non-graspable” TR.

The available observational evidence has shown that TTVIs are feasible in patients with CIED leads, showing comparable short-term outcomes in patients without CIED; however, a minority of them had a defined diagnosis of CIED-related TR [98,99,100]. The VIVID registry has reported some cases of lead dysfunction after valve-in-valve or valve-in-ring procedures [101], while Lurz et al. noted the need to increase the threshold in three cases of T-TEER [99]. Indeed, the risk of lead failure after lead “jailing” or “entrapment” has yet to be elucidated. The risk of lead dysfunction after jailing against the native annulus—in cases of TTVR—is likely less than after entrapment between two artificial rigid annuli, as in valve-in-valve procedures. Moreover, in cases of CIED-related infection, a jailed lead due to a TTVI is a critical hurdle limiting the possibility of an extraction [91]. Finally, the global evaluation of the risk–benefit of lead extraction or transcatheter/surgical procedures in patients with CIED leads should be matter of debate between a multidisciplinary Heart Team, including an electrophysiologist [91].

### 2.6. Access Routes

The evaluation of venous access routes is a pivotal aspect of the success of percutaneous procedures aimed at treating TR. Due to the inherent challenges posed by the complex anatomy of the TV and surrounding structures, selecting the appropriate access site is paramount. Selection of the optimal access route depends on various factors including patient anatomy, procedural requirements, and operator expertise. Among the commonly utilized access routes are the right-sided femoral vein, internal jugular vein, and subclavian vein. Each access point offers its own set of advantages and considerations. Large-bore catheters and delivery systems are used for TTVI, ranging from 22–25 Fr of the commercially available T-TEER systems to 28–34 Fr of most orthotopic valves. For this reason, both orthotopic and heterotopic valve implantation require preprocedural planning with CT [23,24]. Apart from evaluating the venous system, CT sheds light on RA anatomy in terms of the Chiari network, a prominent Eustachian valve, or an unfavorable inferior vena cava (IVC)-RA angle, which may hinder delivery system maneuverability. Specifically, the lateral offset between the IVC and the tricuspid annulus as well as the IVC–tricuspid annulus angle exhibit significant variability among individuals. During tricuspid intervention, a degree of primary and secondary flexion will be required in the delivery system to achieve coaxiality with the tricuspid plane [102]. CT in this context may be useful in anticipating a complex procedure or guiding device choice towards devices with enhanced steering capabilities, as the need for significant secondary flexion can impact primary movements and maneuverability within the RA impeding coaxiality with the tricuspid annular plane [103].

Sometimes, left femoral vein access may help in achieving higher device height from the annular plane if needed [104], whereas the transjugular approach becomes necessary in patients with an occluded IVC system or a caval filter or with some orthotopic valves (such as the LuX-Valve, Jenscare Biotechnology, Ningbo, China). For heterotopic valve implantation with the TricValve system, CT is essential for device sizing because it measures the caval dimensions at various levels: for the superior vena cava (SVC) at the level of the confluence of the innominate veins, at the level of the PA and of the SVC-RA junction and for the IVC at the IVC-RA junction, at the confluence of the hepatic veins and 50 mm below the IVC-RA junction. To avoid hepatic vein obstruction, a minimum distance of 10 mm from the RA-IVC junction to any hepatic vein confluence is necessary [37].

## 3. Key Imaging Factors for Device Selection and Step-by-Step Intraprocedural Guidance

### 3.1. Transcatheter Edge-to-Edge Repair

Transcatheter edge-to-edge repair (TEER) is currently the most commonly performed transcatheter procedure. Two TEER devices have received the CE mark so far: the TriClip G4 (Abbott Structural Heart, Santa Clara, CA, USA) and the PASCAL Precision (Edwards Lifesciences LLC, Irvine, CA, USA). The TriClip is the MitraClip G4 clip-based system with a dedicated delivery system for an improved coaxiality into the right atrium towards the tricuspid valve. The clip is made of two rigid arms of a cobalt–chromium alloy and is based on an active closure mechanism. Four different implant sizes are available (NT, NTW, XT, XTW) on the basis of arm length and width [105]. In the recently reported three-year follow-up of the TRILUMINATE trial, TR reduction achieved at one-year was maintained, with sustained improvements in symptoms, quality of life, and decreased hospitalization rate [106]. The PASCAL system is made of flexible arms of nitinol, a central spacer, and a passive closure mechanism. Two different implant sizes are present (P10 and ACE) [105]. The one-year outcomes of TEER with the PASCAL system show an excellent safety profile with similar benefits for patients as compared to the TriClip [107]. Importantly, both commercially available TEER systems show low complication rates and are probably the least invasive option among the various TTVI techniques, making them ideal for the elder and frail TR population, whenever feasible.

The use of a TEER procedure for TV requires the following conditions: a “graspable” coaptation gap, adequate leaflet length and tissue quality in the grasping target, and an adequate TEE imaging of the tricuspid valve [23]. A simple five-parameter anatomic score evaluating the procedural complexity of T-TEER has been recently developed and externally validated to predict procedural success [108]. The five parameters derived to differentiate between a straightforward and a complex procedure were septo-lateral coaptation gap (0–5 mm vs. ≥6 mm), chordal structure density (modest vs. high), TR jet location (anteroseptal/central vs. posteroseptal/anteroposterior/diffuse), en-face TR jet morphology (oval/linear vs. star-shaped), and TEE image quality (good vs. limited).

**Figure 6:**
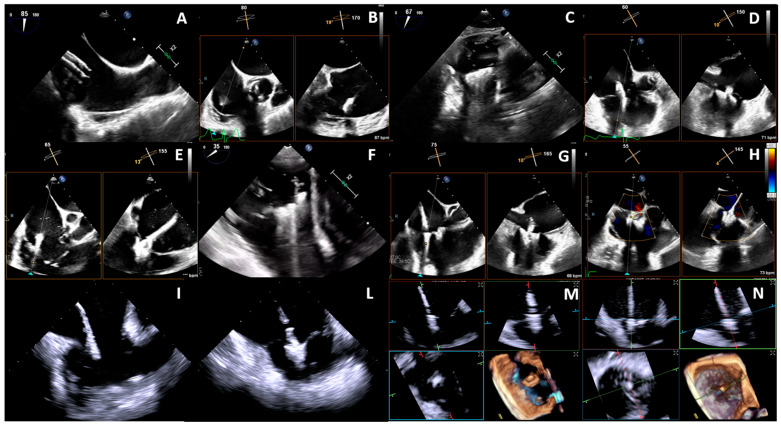
Intraprocedural guidance of T-TEER with TEE (**A**–**H**) or ICE (**I,L**–**N**). See text for details.

The main TEE views for the intraprocedural guidance of TEER are the mid-esophageal inflow–outflow view and the transgastric TV short-axis view (Figure 6A–H). The first procedural step is the insertion of the guide catheter and delivery system into the RA with subsequent exposure of the device. The mid-esophageal bicaval and modified bicaval views (with TV in view) are generally used to guide this initial step (Figure 6A). The clip steering towards the TV plane and the lesion target and the axial alignment are then guided with mid-esophageal inflow–outflow view and biplane-derived 4 chamber views or transgastric views (a TV short axis with a biplane-derived long axis) (Figure 6B). The transgastric TV short-axis view is also useful for clip clocking (or alignment to the coaptation line) (Figure 6C); however, a multiplanar reconstruction of a 3D dataset acquired from a mid-esophageal view may compensate for suboptimal transgastric windows. The fundamental step of leaflet grasping may be guided with trans-esophageal inflow–outflow and a biplane-derived four-chamber view or a transgastric TV short-axis view (Figure 6E,F). The inflow–outflow view is generally preferred due to the possibility of directly assessing the leaflet insertion. The transgastric TV short-axis view, instead, permits only a visual appreciation of the indirect signs of an adequate leaflet grasping (restriction of leaflet motion and clip stability on 2D imaging, adequate tissue bridge on 3D imaging, and TR reduction with color-Doppler). The same steps are repeated in case of positioning of other clips (Figure 6G,H). Finally, a multilevel and multiwindow assessment of clip stability and TR reduction is needed before and after every device deployment. In cases of suboptimal or poor TEE windows, the use of ICE is nowadays a feasible and safe alternative (Figure 6I,L–N) [109]. Noteworthy, the fluoroscopy has a role in the device navigation throughout the TEER procedure, mainly through two perpendicular views: the left anterior oblique caudal view, coinciding with the transgastric short axis view of the TV or a 3D en-face view, and the right oblique caudal view, which corresponds to a transgastric long axis 2-chamber view [23,110].

### 3.2. Orthotopic Valve Replacement

#### 3.2.1. The Evoque System

**Figure 7:**
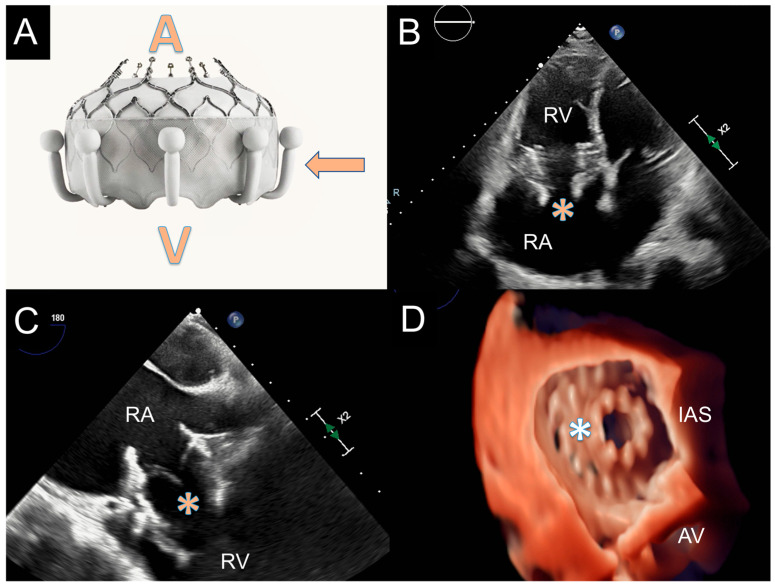
Evoque valve for TTVR. Panel (**A**). The Evoque valve. Atrial side (A) and ventricular side (V) of the valve, the skirt with nine anchors is indicated by the arrow. Panel (**B**). Evoque valve as it appears at TTE (*) in RV-focused 4 chamber view. Panel (**C**,**D**): TEE in mid-esophageal view (Panel (**C**)) and 3D view of the valve (*) from RA side (Panel (**D**)). AV: aortic valve; IAS: interatrial septum; RA: right atrium; RV: right ventricle.

The Evoque Valve (Edwards Lifescience, Irvine, CA, USA) is a TTVR system designed to specifically treat TR [111]. It is made by a self-expandible nitinol frame designed to self-adapt to the tricuspid annulus shape, leaflets made of bovine pericardial tissue and the outer part of the valve covered by an intra-annular sealing skirt characterized by nine anchors (Figure 7). The role of the anchors is to provide leaflets engagement and stabilize the valve into the subvalvular apparatus. The valve is currently available in three sizes (44–48–52 mm) and all sizes are compatible with the 28 Fr delivery system.

The TRISCEND trial [111] launched the Evoque Valve in the European Scenario in the late November of 2023 and it is, to-date, the first transcatheter TV approved for clinical use. This prospective, multicentre, single-arm trial showed a device success of 94.4% with a reduction of TR to mild or less in 97.6% of cases. One-year mortality was low as compared to other transcatheter therapies (9.4% at 1 year) with a composite major adverse cardiac event rate of about 30%, mainly due to severe bleeding (25.5% at 1 year) and new pacemaker implantation (13.3% at 30 days). The estimated 74.9% reduction in heart failure hospitalization is an important result that needs to be confirmed in further randomized trials (the ongoing TRISCEND II) [112]. Even if only one-year follow-up of TRISCEND trial has been published [111], early data of 2-year follow-up are showing similar results [113].

Preprocedural screening is crucial to select patients eligible to treatment and it consists of TTE, TEE and contrast cardiac and vascular CT. TTE is the first screening step and it is mandatory to assess TR etiology, RV function and exclude relevant left ventricular diseases. The main role of TEE is to confirm TR etiology, annular dimensions and to verify the image quality of each TEE view needed for the intraprocedural guidance. CT is the gold standard to assess annular dimensions, to study the subvalvular apparatus and venous access routes.

Beyond clinical evaluation, that is crucial to evaluate the utility of TTVR, there are some key points that need to be assessed to check the eligibility of each patient.

**Figure 8:**
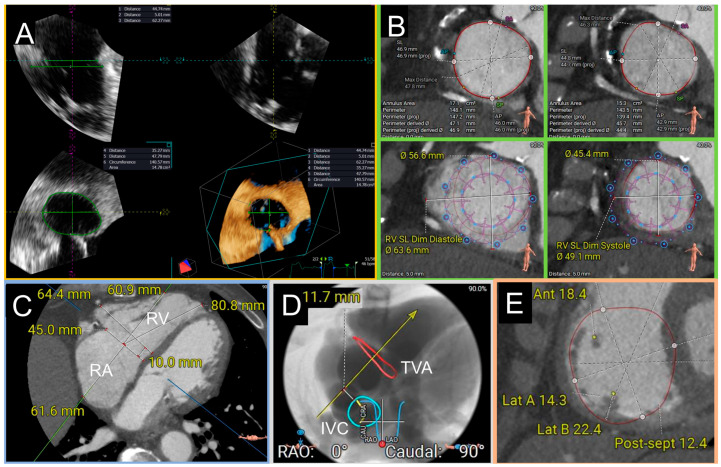
Screening for TTVR, key imaging features. Panel (**A**): TEE 3D volume of TV to assess TV annulus (diameters and perimeter) and basal RV diameter. Panel (**B**): CT evaluation of TV annulus (upper panels) in diastole (panel (**B**) top left) and systole (panel (**B**) top right), and RV at basal level in diastole (bottom left) and systole (bottom right); Evoque anchors are represented in light blue. Panel (**C**): working room assessment; RA and RV length are measured from a reformatted 4 chamber view. Panel (**D**): fluoroscopic reconstruction of implant projection derived from CT images; the distance between inferior vena cava and TVA centerline is measured; in this case it was 11.7 mm (>25 mm is considered a cut-off for non-feasibility). Panel (**E**): contrast CT, evaluation of position and distance of papillary muscles. CT computed tomography; IVC: inferior vena cava; RA: right atrium; RV: right ventricle; TEE: transesophageal echocardiography; TTVR: transcatheter tricuspid valve replacement; TV: tricuspid valve; TVA: tricuspid valve annulus.

– RV function: albeit there is no specific cut-off, severe RV dysfunction was an exclusion criterion in TRISCEND trial [111]. Moreover, at one year follow-up RV systolic function parameters decreased, including RV-FAC (−8.4 ± 13.8%, *p* < 0.001) and TAPSE (−2.8 ± 6.5 mm, *p* = 0.006). It can be reasonable to decide not to treat patients with severely impaired RV function, in order to avoid a futile procedure. The TVARC [32] suggests to use specific cut-offs to define severe RV dysfunction (TAPSE < 10 mm, FAC < 30%, 3D RVEF < 35%), but prospective long-term follow-up studies are needed to validate these cut-offs in the setting of TTVR.
– TR etiology: classical indications for TTVR are TR patients unsuitable for TEER; thus, in clinical practice, it is very common to see patients with extreme TV anatomies (wide coaptation gap, very large annular dimensions, CIED leads, massive and torrential TR). In the TRISCEND trial, 68.2% of TR were functional (atrial or ventricular forms), 9.7% were primary, 14.2% mixed and 2.8% CIED-related [111]. While atrial functional TR usually do not have challenging anatomies, ventricular forms need to be carefully assessed: annular size can be bigger, and valve leaflets can be characterized by tethering or plastering. High tenting height (>15 mm in systole) or leaflet plastering (a distance between leaflets and RV wall < 4 mm in diastole) can affect leaflets engagement and lead to procedural failure. On the other side, in primary TR due to leaflets prolapse or flail, the excessive leaflets mobility can complicate the procedure. If the prolapse/flail is localized, the case can be considered suitable; in the presence of wide septal prolapse/flail (involving more than a half of the leaflets) or lateral leaflets prolapse/flail (>1/3 of valve circumference) procedural success can be significantly compromised. Rheumatic and carcinoid disease induce valve fibrosis reducing systolic-diastolic leaflets movements; the presence of a completely “frozen valve” in diastole, like it is frequently observed in advanced carcinoid syndrome, can prevent leaflets capture and valve anchoring. In the TRISCEND trial about 3% of patients had CIED leads; after TTVR no leads dysfunction or worse outcomes were observed [111]. The presence of CIED leads is not a contraindication for TTVR. However, a comprehensive analysis of leaflet-lead interaction with multimodality imaging (TTE, TEE, CT) is pivotal [114]. In general, if there is an adhesion of CIED-lead with a leaflet, there may be an interference with Evoque valve deployment, but every case must be analyzed individually to ensure feasibility and to predict adequate procedural results.
– Annular sizing: the main reason for screening failure is represented by excessive annular dimensions [111]. With TEE, a perimeter > 160 mm or a 2D diameter > 60 mm are considered highly predictive of screening failure at CT due to excessive annular size. The nominal sizes of the Evoque valve are based on the outer frame diameter of the valve; these diameters are compared to annular dimensions calculated at CT using the projected perimeter derived diameter (PDD, Figure 8). Valve sizing is not only based on pure annular dimensions, but also on the degree of annular oversizing. Annular oversizing is expressed as “[(valve size − PDD)/PDD] × 100” and positive oversizing is identified when the valve is bigger than the native annulus. A positive oversizing is needed to ensure adequate valve sealing. The degree of oversizing is also calculated at the ventricular basal level (and in this case the outer dimensions at the anchors level is used) and its presence is considered a useful retention mechanism.
– Right chambers size: RA and RV represent the working room of the TTVR procedure; adequate chamber size is needed to manipulate the system and implant the valve. The anatomical working room is generally assessed using a contrast CT scan in diastolic and systolic phases. Using a reformatted 4 chamber view, RA height from the annulus to atrial roof, RV length from the annulus to the apex and the relationship between IVC and TV annulus are assessed [115]. The delivery system must have enough space to orientate and implant the valve without any interference with the surrounding structures. Additionally, CT scan is used to assess the position and the distance of papillary muscles relatively to the annulus: from one side papillary muscles can interfere with valve positioning if they are too close to the valve; from the other side, they can be considered another retention mechanism, thus they can stabilize the ventricular side of the valve after deployment (Figure 8).

#### 3.2.2. The Cardiovalve System

The Cardiovalve (Venus Medical) has three bovine pericardial leaflets and was designed to replace the native TV function. Leaflets are mounted on a structure made of a dual self-expandable nitinol frame with two distinct atrial and ventricular aspects welded together. The prosthesis has a dacron-covered flange for improved sealing and 24 grasping points for a firm anchoring in the subvalvular portion. The valve is delivered through a 32 Fr transfemoral approach with a new dedicated delivery system to facilitate steering maneuvers [116].

**Figure 9:**
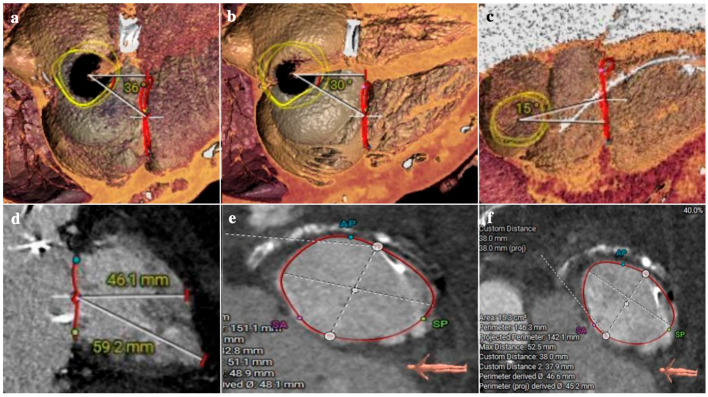
Cardiac CT screening of candidate patient for Cardiovalve implantation: different take-off of the IVC and relationship between IVC and tricuspid annulus (**a**–**c**). Measurement of the distance between the tricuspid annular plane and the free-wall of the RV (**d**); systolic and diastolic dimensions of the annulus together with the evaluation of minimal and maximum diameter (**e**,**f**). CT: computed tomography; IVC: inferior vena cava; RV: right ventricle.

The feasibility of the implant is assessed with a full-cycle cardiac CT scan to provide the following measures both in systolic and diastolic phase (Figure 9):

– RA height: the height is calculated from the TV plane. The minimum cutoff is 60 mm to make the valve positioning feasible and to minimize the risk of complications during the advancement of the delivery system.
– RV length: the minimal distance between the apex of the RV and the tricuspid annular plane is 45 mm in diastole. Below the proposed cutoff, diving of the valve in the ventricle is not feasible.
– IVC offset and top distance: describes the distance and angular relationship between the entry point of the IVC into the RA and the TV annulus. This anatomical detail is crucial for the precise alignment of the delivery system with the TV annulus, for the trajectory and final positioning of the device. An offset less than 20 degree in diastole is usually favourable. The IVC top distance refers to the vertical distance from the superior aspect (or top) of the IVC to the TV annulus and it should be at least 50 mm to have a correct insertion depth and trajectory.
– Annulus diameters: minimal annulus diameter should be above 36 mm. Moreover, the shape of the annulus should not be too oval to avoid device mismatch. Cardiovalve has 3 different sizes that cover a broad range of diameters (45–55 mm).

The Cardiovalve system is delivered through a transfemoral approach. The initial evaluation should be focused on valve steering towards the valvular plane. Once the valve is in position, 3D views and transgastric imaging must verify the correct central positioning of the prosthesis in the tricuspid space. Once opened, the valve exposes anchors which are dived in the subvalvular space. Once the correct positioning of the prosthesis is confirmed, the whole system is pulled back to engage the native ventricular aspect of the leaflets. The correct engagement of leaflets is verified using multiplanar reconstruction and biplane views with verification of each anchor; restricted motion of the leaflets is used as an indirect sign of correct valve positioning. Once the valve is seated nicely and anchors are engaged, a two steps release is performed. The valve starts functioning immediately. TEE imaging immediately assesses leaflets opening, leaks and any possible procedural complication [21].

### 3.3. Heterotopic Valve Replacement

Heterotopic valve replacement is a possible percutaneous treatment to mitigate symptoms and signs of TR. The only available option is the TricValve system (P+F Products + Features GmbH), which consists of two self-expanding valves delivered in the SVC and IVC, separately [117]. New devices such as Trillium and UNICA are novel single-stent, double-valve, heterotopic, cross-caval systems under clinical development [118,119].

**Figure 10:**
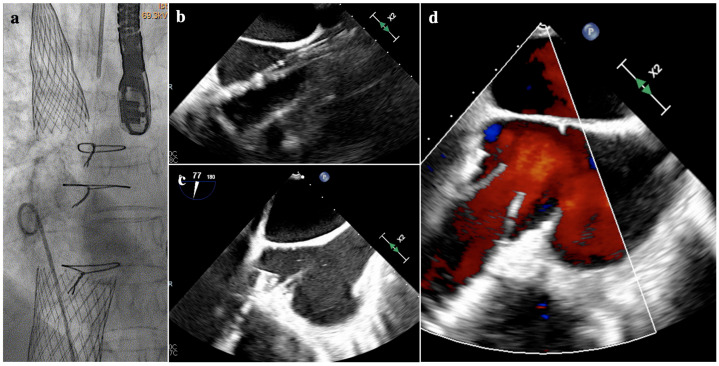
An example of TricValve implantation with a 29 mm prosthesis in the SVC and 35 mm prosthesis in the IVC (**a**). TEE monitoring can be challenging for the superior prosthesis, and visualization is often suboptimal (**b**). The TEE can easily visualize inferior prosthesis delivery (**c**) and evaluate valve function and positioning (**d**). IVC: inferior vena cava; SVC: superior vena cava; TEE: transesophageal echocardiography.

Patient selection for TricValve is based upon clinical, echocardiographic and cardiac CT scan criteria. Baseline echocardiography should exclude significant RV failure (defined as TAPSE ≤ 13 mmHg) and significant pulmonary hypertension (PAPs > 65 mmHg). A CT scan is mandatory to verify the feasibility of the implant. Key measures are the diameters of the SVC and IVC at different levels, the SVC- and IVC-RA junction. Most of the procedures are performed in conscious sedation and without need for TEE imaging. When available, TEE can hardly give any information on SVC delivery because of the poor visualization [4]. TEE and/or TTE in subcostal view are utilized to assess valve protrusion in the RA. Valvular frames should protrude no more than 15 mm in the RA. After valve release in the IVC, color-Doppler imaging gives details about leaks and flow in the superior hepatic vein; a careful assessment of RV function and contractility is mandatory after the release of both valves (Figure 10).

### 3.4. Transcatheter Tricuspid Valve-in-Valve Replacement

Among the spectrum of transcatheter procedures on the TV, a sizeable number of valve-in-valve (VIV) and valve-in-ring (VIR) procedures are being performed on failing bioprostheses and previous surgical repairs in order to avoid high-risk re-do surgeries [120]. Akin to procedures performed on the aortic, mitral or pulmonary valves, the previously implanted prosthesis functions as a stable landing zone for a new balloon-expandable valve. The largest series reporting on tricuspid VIV and VIR is the VIVID registry, which showed encouraging short- and mid-term follow-up data [121,122]. The investigators reported mixed stenosis and regurgitation as the prevalent mechanism of failure (47%), followed by stenosis (29%) and regurgitation (24%). Procedural success was achieved in 99% of cases using valves such as the Sapien XT, Sapien 3, and Melody. Notably, tricuspid VIV and VIR are feasible even in cases of CIED leads transversing the surgical prosthesis [101], although lead entrapment may pose future challenges. The procedure is safe and leads to significant improvement in tricuspid gradients and the degree of regurgitation [122]. Paravalvular leaks are an issue, especially in cases of VIR procedures, due to the limited ability of some surgical rings to become circular when accommodating the new valve.

Intraprocedural imaging is required in the form of TEE (or TTE) and fluoroscopy. Femoral or internal jugular vein access can be used. The annular plane and a coplanar fluoroscopic plane are identified by aligning the radiopaque elements/sewing ring of the bioprosthetic valve. RV angiography may be necessary in case of valves or rings with a paucity of radiopaque elements. The degenerated bioprosthetic valve or ring is crossed with a stiff wire left in the RV (which is the preferred position for better coaxial alignment) or in the PA. Due to RA dilatation, crossing may be easier by using a steerable sheath. Pre-dilatation may be performed. The delivery system is then advanced, and the valve is deployed (with or without rapid pacing) under fluoroscopic and echo guidance. Post-dilatation is similarly optional. Finally, echocardiography is used to evaluate the position and anchoring of the new valve and the presence of paravalvular leaks and transvalvular gradients.

## 4. Conclusions

The field of percutaneous repair and replacement techniques for TR is relentlessly growing, as new devices enter the market and established ones become more sophisticated. Optimal patient and device selection, guided by refinements in cardiac imaging and tailored to individual anatomy and clinical characteristics, will be decisive in optimizing acute procedural success, durability, and long-term outcomes, alongside growing experience with the single devices.

## Figures and Tables

**Table 1:** Etiologic classification of tricuspid regurgitation. Abbreviations: TR: tricuspid regurgitation; CIED: cardiac implantable electronic device; RV: right ventricular; HFpEF: heart failure with preserved ejection fraction; RA: right atrium.

	Primary TR	Secondary Ventricular TR	Secondary Atrial TR	CIED-Related TR
	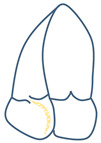	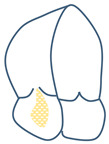	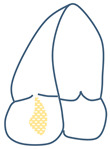	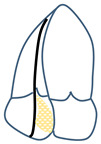
**Prevalence**	10–15%	55–75%	10–15%	5–15%
**Leaflet morphology**	Abnormal	Normal but tethered	Normal	Variable
**Etiology**	Prolapse Flail Rheumatic Carcinoid Endocarditis Post-attinic Trauma Congenital Iatrogenic	Left heart disease Pulmonary hypertension RV disease	Atrial fibrillation HFpEF	Lead-induced impingement, entrapment, tear or perforation of a leaflet; RV dyssynchrony or dysfunction
**Pathophysiology**	Carpentier I Carpentier IIIA	Carpentier IIIB	Carpentier I	Carpentier I, II, IIIA, IIIB
**Imaging** **features**	Coaptation gap, leaflet restriction, leaflet perforation	Leaflet tethering, RV dilatation/dysfunction	Annular dilatation, RA dilatation	Position and relationship of the lead to the leaflets (3D echocardiography)

**Table 2:** Contemporary grading of TR severity. Abbreviations: VC: vena contracta; EROA-PISA: Effective regurgitant orifice area–proximal isovelocity surface area; VCA-3D: vena contracta area, three-dimensional.

Parameter	Mild	Moderate		Severe	Massive	Torrential
**VC biplane**	<3 mm	3–6.9 mm		7–13 mm	14–20 mm	≥21 mm
**EROA-PISA**	<20 mm^2^	20–39 mm^2^		40–59 mm^2^	60–79 mm^2^	≥80 mm^2^
		**Mild-to-moderate**	**Moderate-to-severe**			
		20–29 mm^2^	30–39 mm^2^			
**VCA-3D**	-	-		75–94 mm^2^	95–114 mm^2^	≥115 mm^2^

**Table 3:** Useful parameters for assessing the right heart and the pulmonary vasculature through different techniques. TAPSE (tricuspid annular plane systolic excursion); FAC (fractional area change); RVEF (right ventricular ejection fraction); RVEDV (right ventricular end-diastolic volume); RVEDVi (right ventricular end-diastolic volume indexed); RVESV (right ventricular end-systolic volume); GLS (global longitudinal strain); FWLS (free wall longitudinal strain); TAPSE/PAPs (tricuspid annular plane systolic excursion/pulmonary artery systolic pressure); RCA (right coronary artery); right atrial pressure (RAP); right ventricular systolic pressure (RVSP); right ventricular diastolic pressure (RVDP); pulmonary artery systolic pressure (PAPs); pulmonary artery diastolic pressure (PAPd); pulmonary capillary wedge pressure (PCWP); cardiac output (CO); mean pulmonary artery pressure (PAPm); trans-pulmonary pressure gradient (TPG); cardiac index (CI); stroke volume (SV); stroke volume index (SVI); systemic vascular resistance (SVR); systemic vascular resistance index (SVRI); pulmonary vascular resistance (PVR); pulmonary vascular resistance index (PVRI); pulmonary artery pulsatility index (PAPi); body surface area (BSA); Wood units (WU); right ventricular stroke work (RVSW); LLN (lower limit of normal); ULN (upper limit of normal); CT: computer tomography; CMR: cardiac magnetic resonance; TTE: trans-thoracic echocardiography. Arterial oxygen saturation (SaO2) should be acquired by arterial blood gas measurement; mixed venous oxygen saturation (SvO2) should be acquired by mixed venous gas from a pulmonary artery catheter.

TTE ^a^			CARDIAC CT ^c^		CMR ^e^		RHC ^f^		
**Longitudinal systolic function**	Mean ± SD	LLN	**Distance from RCA to** **TV apparatus**		RVEDV, mL	176–244	RAP	0–6 mmHg	
TAPSE, mm	21 ± 3.8	15.2	*At the level of anterior tricuspid leaflet*		RVESV, mL	48–123	RVSP	15–25 mmHg	
S’	13 ± 2.3	9.4	Transverse distance to RCA, mm	8.8 ± 4.5	RVEF, %	46–72	RVDP	8–15 mmHg	
**Circumferential systolic function**			Vertical distance to RCA, mm	4.4 ± 1.8			PAPs	15–25 mmHg	
RV FAC, %	42 ± 4.3	35.3	*At the level of posterior tricuspid leaflet*				PAPd	8–15 mmHg	
**RV size (RV apical focused view)**		ULN	Transverse distance to RCA, mm	3.6 ± 3.4			PCWP	6–12 mmHg	
RV basal diameter, mm	32.8 ± 5.3	44.2	Vertical distance to RCA, mm	4.6 ± 2.3			**Derived Measures**	**Normal values**	**Formulas**
RV mid diameter, mm	26.4 ± 5.7	38.6	**Tricuspid valve annulus**				CO (Fick)	4–8 L/min	125 × BSA/[Hb × 1.36 × (SaO2 − SvO2)]
RV length, mm	73.7 ± 8.7	91.8	Antero-posterior diameter, mm	44.5 ± 5.1			PAPm	10–20 mmHg	[PAPs + (2 × PAPd)]/3
Tricuspid annulus, mm	28.6 ± 5.1	39.4	Septal-lateral diameter, mm	37.3 ± 5.4			TPG	<13 mmHg	PAPm − PCWP
**3D**	Normal value		Perimeter, mm	132.4 ± 5.3			CI	2.5–4 L (min × m^2^)	CO/BSA
RVEF, %	56–71		Area, mm^2^	1297 ± 297			SV	60–100 mL/beat	CO/HR × 1000
RVEDV, mL/m^2^	43–66		Distance from anulus to RV, mm	70 ± 10			SVI	33–47 mL (m^2^ × beat)	CI/HR × 1000
RVESV, mL/m^2^	12–28		**Function ^d^**	**Normal value**			SVR	800–1200 dynes-sec/cm^5^	80 × (MAP − RAP)/CO
**Strain**		LLN	RVEDV, mL	175 ± 48			SVRI	1970–2390 dynes-sec/cm^5^/m^2^	80 × (MAP − RAP)/CI
RV-GLS, %	18–21	<14	RVEDVi, mL/m^2^	93 ± 20			PVR	<3 WU	(PAPm − PCWP)/CO
RV-FWLS, %	20–23	<15	RVESV, mL	82 ± 29			PVRI	255–285 dynes-sec/cm^5^/m^2^	80 × (PAPm − PCWP)/CI
**Ventricular-arterial coupling ^b^**		LLN	RVEF, %	58 ± 8			PAPi	>0.9	(PAPs − PAPd)/RAP
TAPSE/PAPs	0.406	<0.406					RVSW ^g^	7–19 g × m	(PAPm − RAP) × SV × 0.0136

^a^ Adapted from Hahn RT et al. [32]. ^b^ Adapted from Brener MI et al. [73]. ^c^ Adapted from van Rosendael PJ et al. [76]. ^d^ Adapted from Badano et al. [65]. ^e^ Adapted from Kawel-Boehm N et al. [80]. ^f^ Adapted from Del Rio-Pertuz G et al. [84]. ^g^ Adapted from Stocker TJ et al. [87].

## Data Availability

No new data were created or analyzed in this study. Data sharing is not applicable to this article.
